# Effect of Maternal Obesity and Preconceptional Weight Loss on Male and Female Offspring Metabolism and Olfactory Performance in Mice

**DOI:** 10.3390/nu11050948

**Published:** 2019-04-26

**Authors:** Polina E. Panchenko, Marie-Christine Lacroix, Mélanie Jouin, Sarah Voisin, Karine Badonnel, Marion Lemaire, Nicolas Meunier, Sofiane Safi-Stibler, Marie-Annick Persuy, Luc Jouneau, Didier Durieux, Simon Lecoutre, Hélène Jammes, Delphine Rousseau-Ralliard, Christophe Breton, Claudine Junien, Christine Baly, Anne Gabory

**Affiliations:** 1 UMR BDR, INRA, ENVA, Université Paris-Saclay, 78350 Jouy-en-Josas, France; polina.panchenko@bristol.ac.uk (P.E.P.); melanie.jouin@inra.fr (M.J.); sarah.voisin.aeris@gmail.com (S.V.); marionlemaire@laposte.net (M.L.); sofiane.safi-stibler@inra.fr (S.S.-S.); luc.jouneau@inra.fr (L.J.); helene.jammes@inra.fr (H.J.); delphine.rousseau@inra.fr (D.R.-R.); claudine.junien@inra.fr (C.J.); 2 NBO, INRA, Université Paris-Saclay, 78350 Jouy-en-Josas, France; mclacroixkann@gmail.com (M.-C.L.); karine.badonnel@inra.fr (K.B.); nicolas.meunier@inra.fr (N.M.); marie-annick.persuy@inra.fr (M.-A.P.); didier.durieux@inra.fr (D.D.); christine.baly@inra.fr (C.B.); 3 Équipe Malnutrition Maternelle et Programmation des Maladies Métaboliques, EA4489, Université de Lille, 59000 Lille, France; 23simon.lec@gmail.com (S.L.); christophe.breton@univ-lille1.fr (C.B.)

**Keywords:** obesity, preconceptional weight loss, olfaction, programming, DOHaD, metabolism

## Abstract

According to the “developmental origins of health and disease” (DOHaD) concept, maternal obesity predisposes the offspring to non-communicable diseases in adulthood. While a preconceptional weight loss (WL) is recommended for obese women, its benefits on the offspring have been poorly addressed. We evaluated whether preconceptional WL was able to reverse the adverse effects of maternal obesity in a mouse model, exhibiting a modification of foetal growth and of the expression of genes encoding epigenetic modifiers in liver and placenta. We tracked metabolic and olfactory behavioural trajectories of offspring born to control, obese or WL mothers. After weaning, the offspring were either put on a control diet (CD) or a high-fat (HFD). After only few weeks of HFD, the offspring developed obesity, metabolic alterations and olfactory impairments, independently of maternal context. However, male offspring born to obese mother gained even more weight under HFD than their counterparts born to lean mothers. Preconceptional WL normalized the offspring metabolic phenotypes but had unexpected effects on olfactory performance: a reduction in olfactory sensitivity, along with a lack of fasting-induced, olfactory-based motivation. Our results confirm the benefits of maternal preconceptional WL for male offspring metabolic health but highlight some possible adverse outcomes on olfactory-based behaviours.

## 1. Introduction

Obesity is the 5th largest risk factor for mortality and is associated with severe conditions such as cardiovascular diseases, stroke, type-2 diabetes, dyslipidaemia, sleep disorders, hepatic steatosis, osteoarthritis, cancer and neurocognitive defects [1,2]. In addition, obesity-induced olfactory impairments have been described in human [3] and in several animal models [4,5,6], pointing at a tight link between olfaction and metabolism imbalance (reviewed in [7]). An excessive body mass index (BMI, weight/height^2^) is considered a risk factor for loss of olfaction and eating disorders [8,9,10].

The “developmental origins of health and disease” (DOHaD) concept states that obese women give their children an increased risk of developing non-communicable diseases (NCDs) in adulthood. Early exposure to various exogenous or endogenous changes during preconceptional period, gestation, lactation or childhood and adolescence affect long-term health [11,12,13]. It is known that maternal nutrition affects the offspring’s non-homeostatic regulation of food intake, but its consequences on offspring olfactory performance have poorly been investigated in the DOHaD context (see [7] for review). Yet, the perinatal period is crucial for the development of the olfactory system that is already functional in utero and that displays anatomical and functional neuroplasticity to achieve its maturity at the end of the third postnatal week in rodents [14]. The olfactory system is thus likely to be affected by maternal diet with consequences on food intake behavior, associated or not with impairments in olfactory sensitivity. However, curiously, handful of animal studies have examined the consequences of maternal energy deficit or surplus on offspring’s olfaction. Undernourishment throughout gestation and suckling results in functional immaturity of the offspring’s olfactory system, leading to both short- and long-term deficiencies in odour discrimination [15,16]. Moderate maternal caloric restriction during pregnancy led to a sex-specific decrease of an endocannabinoid neurotransmitter in the main olfactory bulb of female offspring [17]. To our knowledge, very few studies were done in obese context. Very recently, a deleterious effect of a maternal high-fat and high-sugar diet on male offspring’s olfactory performances at weaning has been demonstrated [18]. It is thus highly conceivable that maternal nutrition and weight changes might influence olfactory-based behaviour in offspring, potentially impacting their long-term food preferences and intake, as previously reported [19,20].

Obesity is also associated with reproductive issues such as infertility, obstetrical complications, birth defects and stillbirth [21]. When limited weight gain during pregnancy was advised to obese mothers to improve these outcomes, there were only small effects on foetal growth and metabolic outcomes [22,23]. A preconceptional weight loss (WL) is now widely recommended to obese women, improves fertility and reduces the occurrence of metabolic complications [24]. However, whereas some studies demonstrate a positive impact [25,26,27,28], other studies indicate potential deleterious effects [28,29,30]. Interestingly, none of these studies followed up the metabolic development of these children and adolescents and additional studies are therefore needed to reveal the long-term health profiles of offspring born to obese mothers who lost weight prior to conception [31,32]. In two rat models, a nutritional intervention in obese dams was beneficial to the offspring, even if all parameters were not normalized [33,34]. In sheep, a strict nutritional intervention before mating had long-term benefits on offspring weight gain but deleterious effects on offspring stress response and glucose metabolism [35,36]. WL following bariatric surgery in humans [37,38,39], or diet reversal in rodents [5], showed mixed results on olfactory performance, suggesting that alterations to olfaction are difficult to reverse.

Further work is required to determine the effect of maternal preconceptional BMI and WL on metabolic outcomes and olfactory behaviour related to food intake in the offspring. To our knowledge, no study has investigated the contribution of either maternal obesity linked to high fat consumption or preconceptional WL on the preservation, improvement or impairment of olfactory performance in the offspring at different ages, and with a focus on sex differences. Here, we evaluated whether a preconceptional WL may be used as a therapeutic tool to reverse the adverse effects caused by a gestational obesity (OB) on offspring’s phenotype, using a previously described animal model developed in our laboratory [40]. Metabolic and behavioural trajectories of male and female offspring until adulthood were followed, under two post-weaning macronutrient options: a control (CD) or a high-fat diet (HFD). We confirmed the main effect of post-weaning diet on the offspring metabolism and olfaction. However, we showed that maternal obesity and weight loss also influence the next generation phenotype, with sex-specificities. Male offspring born to obese mother presented a worsened diet-induced obesity than their counterparts born to lean mothers. Preconceptional WL normalized the offspring metabolic phenotypes but had unexpected effects on olfactory performance with a reduction in olfactory sensitivity, along with a lack of fasting-induced, olfactory-based motivation.

## 2. Materials and Methods

### 2.1. Animal Experiment Procedures

The COMETHEA ethical committee (Comité d’éthique pour l’expérimentation animale), registered with the Comité National de Réflexion Ethique sur l’Expérimentation Animale under the n°45, approved this protocol (visa 12/062), in accordance with European Union (Directive 2010/63/EU, 22 September 2010) legislation and the National charter on the ethics of animal experimentation.

Four-week-old female and 7-week-old male C57Bl/6J mice were purchased from Harlan Laboratory (Venray, Netherlands) and housed in IERP animal holding facilities (Unité d’Infectiologie Expérimentale des Rongeurs et Poissons; INRA, Jouy-en-Josas, France) at controlled temperature (22 ± 2 °C) with a 12 h light/12 h dark cycle. Mice had ad libitum access to water and food and paper towel was provided for nest building. After one week of adaptation, mice were placed in individual cages and randomly assigned to either the high-fat diet (HFD) or the matched control diet (CD, Table 1). Food was purchased in pellet form, stored at 4 °C and replaced weekly to prevent degradation. Macronutrient and micronutrients (vitamins, salts, etc.) in these diets are balanced to the caloric content, because rodents adjust their caloric intake. Generations F0 and F1 mice were weighted and food intake was recorded twice a week. Relative caloric intake (kcal/kg of body weight/day) was calculated for each mouse.

**Figure 1:**
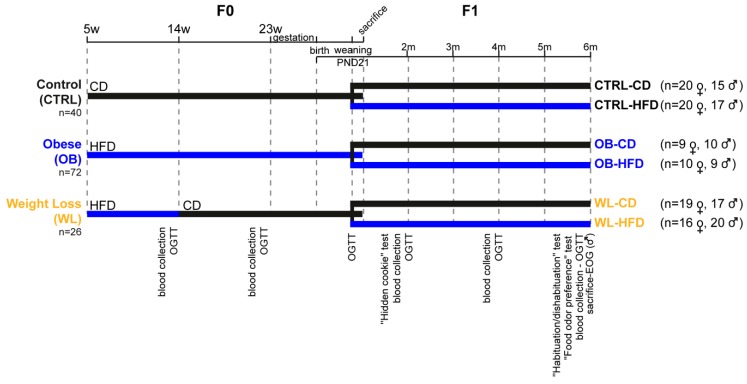
Experimental protocol for studying the effects of maternal periconceptional and postweaning diets on offspring. Female C57BL/6J F0 mice were fed a high-fat (HFD, blue bars) or a control (CD, black bars) diet. After 2 months, a subset of HFD mice was assigned to CD for the next 2 months in order to induce a weight loss (WL group). The remaining mice stayed on HFD to induce a chronic obesity (OB group). Control mice (CTRL) received CD for the 4 months. Females were mated and remained on their experimental diets through pregnancy and lactation. At weaning, male and female offspring were randomly assigned onto CD or HFD: 6 F1 groups were thus obtained. All mice were monitored for body weight and food intake. F0 were sacrificed one week after weaning and F1 were sacrificed at 6 months of age. The schedule of blood collection (leptin and cholesterol measures), metabolic (oral glucose tolerance test, OGTT) and olfactory behavioural tests (“hidden cookie”, “habituation/dishabituation”, “food odour preference” tests and electro-olfactogram, EOG) is indicated. *n*, individual number per group; w, the week of age; m, the month of age; PND, post-natal day.

We used two different season-matched F0 cohorts. From 5 weeks of age, mice of the control group received CD for the 4 months in preconception period (CTRL *n* = 40; Figure 1). The others received the HFD (*n* = 98). At 14 weeks, after 2 months of HFD, mice with a weight above the threshold of mean CTRL weight +2 standard deviations, were considered to be obese [41] and one female out of two was assigned to CD for the next two months of experimental procedure (Figure 1) in order to induce a weight loss (WL group, *n* = 26). The remaining mice stayed on HFD to develop a chronic obesity (OB group, *n* = 72). Measurements of fasting cholesterolemia, glycaemia, insulinemia, leptinemia and oral glucose tolerance test (OGTT) were performed at the age of 13 and 22 weeks. From 23 weeks of age, females were mated with chow-fed C57BL/6J males (#801030 RM3A; Special diets services, Witham, Essex, UK) for one night. If no vaginal plug was observed, females were mated with another male according to their estrus cycle. The reproduction period lasted for 6 weeks. Females remained on their experimental diets throughout pregnancy and lactation. At E16.5, litter was last changed in the cage and nestlets were provided. All female mice gave birth by spontaneous vaginal delivery.

At postnatal day (PND) 1, litters were reduced to six pups when necessary. Litter was changed in the cage at PND7 and F1 were weighed every 2 days until PND21. Then, male and female offspring were randomly fed a CD or HFD to obtain 6 F1 groups (Figure 1): CTRL-CD, CTRL-HFD, OB-CD, OB-HFD, WL-CD and WL-HFD. Measurements of fasting cholesterolemia, glycaemia, insulinemia and Oral Glucose Tolerance Tests (OGTT) were performed at weaning and 2, 4 and 6 months. A battery of behavioural tests was conducted in the following order: a hidden cookie test (age: 2 months), a habituation/dishabituation test (5.5 months) and a food odour preference test (6 months, Figure S1, Methods S1). One OB mother and its 4 pups were removed from the experiment since it had lost more than 20% of its weight before the beginning of gestation.

### 2.2. Assessment of Glucose Metabolism

After 6 h fasting (8:00 a.m. to 2:00 p.m.), a bolus of glucose (2 g/kg body weight) was delivered into the stomach of conscious mice using a gavage probe. Glycaemia was measured prior glucose delivery (T0) and at 20, 40, 60, 90 and 120 min. Insulinemia was measured at 0, 20 and 60 min, except for the F1 mice at weaning, for whom only at fasting (T0) insulinemia was measured. The trapezoidal method was used to calculate the area under the curve (AUC) (auc function of the flux package in R) considering only values above the fasting level. Glycaemia from tail vein blood was measured in duplicate using an Accu-Chek Performa blood glucose meter (Roche diagnostics GmbH, Germany). Tail vein blood (30µL) was collected in microvette tubes (Rajouter la ville, Sarstedt, Nümbrecht, Germany), centrifuged for 10 min at 2000 g at 4 °C. Insulinemia was measured with Mouse Ultrasensitive Insulin ELISA (#80-INSMSU, Alpco, Salem, NH, USA), according to the manufacturer’s instruction. Homeostatic Model Assessment for Insulin Resistance (HOMA-IR) was calculated as: fasting glycaemia (mol/L) × insulinemia (mol/L)/22.4.

### 2.3. Assessment of Lipid Metabolism

At 2:00 p.m., after 6 h fasting, submandibular vein blood (300 µL) was collected from conscious mice in tubes containing 5 U.I. of heparin (Choay heparin, Sanofi-aventis, Paris, France). Samples were centrifuged for 10 min at 1500× *g* at 20 °C, plasma collected and stored at −20 °C. For F0, total cholesterol level was measured by colorimetric dosage on Ortho Vitros Clinical Chemistry System instrument in Ambroise Paré Hospital (Boulogne-Billancourt, France). For F1, cholesterolemia was measured by enzymatic CHOD-PAP (cholesterol oxidase: p-aminophenazone) method (#WCHO100, Sobioda, Montbonnot-Saint-Martin, France). Leptinemia was measured with Leptin Mouse/Rat ELISA (#RD291001200R, BioVendor Laboratory Medicine, Brno, Czech Republic) according to the manufacturer’s guidelines.

### 2.4. Assessment of Body Composition

At 6 months of age, F1 mice were weighed and sacrificed by cervical dislocation after 5–8 h fasting (from 8:00 a.m. to 13:00–16:00 p.m.). The weight of following tissues was recorded: subcutaneous (Sc), perirenal (Pr) and perigonadal (Pg) white adipose tissue (WAT), brown adipose tissue (BAT), liver, kidney and heart. Organ weights are presented relative to body weight (%).

### 2.5. Olfactory Behavioural Experiments

All behavioural tests were performed during the light phase, at 2:00 p.m., with water and food available, except when mentioned. Data were collected by experimenters blind to diet groups.

#### 2.5.1. The Olfactory Sensitivity Test (Hidden Cookie Test)

It was performed as described previously in rats with slight modifications [4]. Mice were familiarized with their own experimental cage (4 cm bedding, 1 h/day) for 3 days. On the fourth day, mice were tested with an unscented square tile hidden under the bedding to evaluate their curiosity and anxiety-based digging behaviours (referenced as experimental day D0). The next four consecutive days and twice a day at 1 h intervals, mice were dropped off in the middle of their experimental cage, where two 5-mm pieces of cheese had been hidden under the bedding in a random corner of the cage, changed at every trial. Time for cheese-retrieving (in seconds) was recorded for 5 min. Cheese was deemed to be found when the mice were holding it or if at least 10% of it was visible. If the cheese was not found, time was considered 300 s and the piece was given to the mouse. All mice consumed the cheese after the first day. The scores of the two daily trials were averaged. Between each trial, animals were put back in their home cages. From D1 to D3, mice were tested in the fed state; on D4, mice were tested after 8 h fasting, first with the unscented tile square, then with the cheese.

#### 2.5.2. Habituation/Discrimination Test

The capacity of mice to discriminate two chemically distinct odorants was measured as described [42], in a large plastic experimental cage (40 cm × 25 cm × 14.5 cm) in which a flat plastic board with four holes were bored at each corner of the board (2 cm diameter, 3 cm from the edge) and left in a one-hole configuration. A glass container was placed under the open hole with a piece of Whatman paper, covered with a half tea-ball metallic protection, so that mice could not touch it. Six-hour fasted mice were familiarized with the experimental setup in a 2 min trial without odorant. The test consisted of successive presentations (2 min each) of a Whatman paper soaked with 60 µL of mineral oil (twice, odourless control), pentanol (0.074% *v*/*v*; four times, habituation), followed by decanal (1.78% *v*/*v*; once, discrimination). The time spent sniffing the hole (in seconds) was manually recorded. Between each presentation, animals returned in their cage for 5 min. Animals that did not display any sniffing during the test were removed from the statistical analysis (4 CTRL-CD, 4 CTRL-HFD, 3 OB-CD, 2 OB-HFD, 4 WL CD and 3 WL-HFD males; 2 CTRL-CD, 2 CTRL-HFD, 2 OB-CD, 1 OB-HFD, 3 WL-CD and 1 WL HFD females).

### 2.6. Electro-Olfactogram (EOG)

EOG recordings were performed from the centre of turbinates IIb and III of the olfactory epithelium in an opened nasal cavity configuration on male mice during light phase (2:00–5:00 p.m.) as already described [43]. Heptaldehyde was tested at serial dilutions ranging from 1:100,000 to 1:1000 in mineral oil (Sigma Aldrich, Saint-Quentin Fallavier, France). Peak amplitude was measured. For organizational reasons, EOG was performed only in males, which presented a more pronounced metabolic phenotype according to maternal group.

### 2.7. Statistics

The data were analysed using the R software. Male and female offspring were tested separately as there was sexual difference for most phenotypes. For all tests, an adjusted *p*-value of *p* < 0.05 was considered to be significant.

For metabolic data, as variances were unequal, data were Boxcox transformed, using the powerTransform and bcPower functions of the car package and analysed with a likelihood-ratio test, using the lrtest function of the lmtest package, and adjusted with the Benjamini and Hochberg (BH) correction for multiple comparisons using the p.adjust function. Linear mixed models were used to model the evolution of metabolic phenotypes with time, using the lmer function of the lme4 package. Estimates of the slopes for each phenotype were reported as “β” and coded as Phenotype = Group + Time + Group × Time+random(ID × Time) and Phenotype = Group + Time + Diet + Diet × Time + Group × Time + Group × Diet × Time + random(ID × Time) for F0 and F1 respectively, where “Group” is the maternal group (CTRL, OB or WL), “Diet” is the F1 post-weaning diet (CD or HFD), “Time” is the timepoint and “random(ID × Time)” is a random intercept and slope component for repeated measures. We used ANOVA to test for differences at each timepoint, using the aov function followed with Tukey’s post hoc test, coded as Phenotype = Group or Phenotype = Group + Diet + Group × Diet for F0 and F1 respectively. Effect size was reported as the proportion R2 of the variance in the phenotype of interest. The PCA function of the FactoMineR package was used in F0 dams.

For olfactory scores, the distribution of the variance was heterogeneous, so nonparametric statistics were performed. N-ways ANOVAs were done using the aovp function of the Permutation Tests for Linear Models (lmperm) package, with factors: Groups × Diet in Figure 4a and Group × Diet × fasting in Figure 4c, Group × Diet × Odorant in Figure 5 (discrimination) and Group × Diet × Odorant dilution in Figure 6; or the nonparametric analysis longitudinal data (nparLD) package [44] with Groups × Diet as whole plot factors in and Days in Figure 4b or Odorant presentations in Figure 5 (habituation) as a subplot. Post-hoc tests (coin package) followed by BH correction were performed: symmetry-test (Figure 4b), Wilcoxon test (Figure 4b and Figure 5) and the two-sample Fisher-Pitman permutation test for comparative analysis of two independent diet group (Figure 4), stratified by dose (Figure 6).

A multiple factor analysis (MFA) was performed in F1 generation on a set of 18 variables regrouped in 3 variable categories (biochemisty, biometry and behaviour), using the FactoMineR package [45]. “Biochemistry” grouped the last measure of fasting glycaemia, insulinaemia, cholesterolemia and leptinemia, and glycemic AUC in OGTT. “Biometry” grouped the organ to body weight ratio for BAT, Pr-WAT, Pg-WAT, Sc-WAT, Total-WAT, Liver, Heart and Kidney, and week-24.5 body weight. “Behaviour” grouped the week-23 relative caloric intake, day-1 hidden-cookie test retrieving time, HFD filled hole preference ratio and EOG at dose 0.001. Hierarchical clustering analysis followed by a χ^2^ and a v.test, which is a normalized difference between the average of the individuals of a class and the general average, corroborated the graphical results. Significance is reached when χ^2^ *p* < 0.05 and v.test > 1.96 in absolute value. In the variables factor map, for a given dimension, the variables most correlated to the dimension are close to the circle. Significance is reached when *r^2^* > 0.6 in absolute value and *p* < 0.05.

## 3. Results

### 3.1. Preconceptional Weight Loss Normalizes the Metabolic Profile of Obese Dams

**Figure 2:**
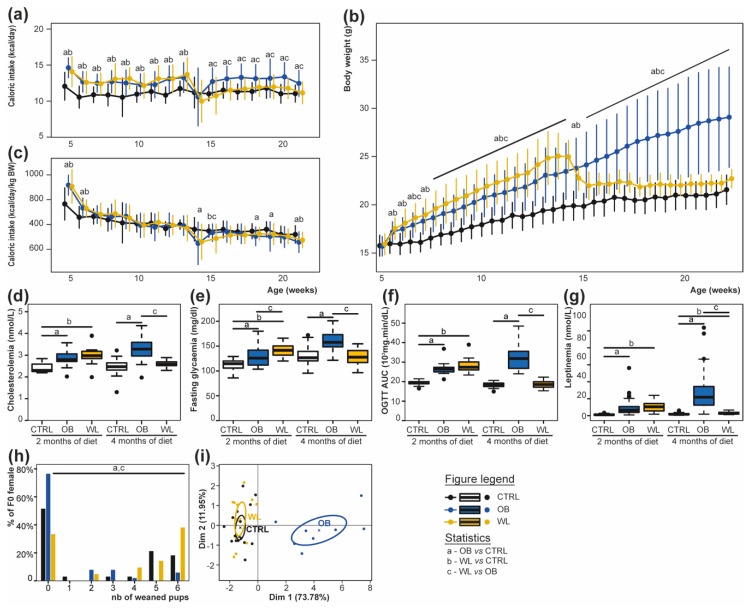
OB dams develop obesity, dyslipidaemia and diabetes and preconceptional WL normalises these parameters. F0 mice were monitored for caloric intake in kcal/day (**a**), body weight (**b**), or kcal/day/kg body weight (**c**), CTRL, *n* = 20–40; OB, *n* = 24–72; and WL, *n* = 12–26, represented as mean ± SD. At 2 and 4 months of preconceptional diet, cholesterolemia (**d**), fasting glycemia (**e**), oral glucose tolerance test (**f**), and leptinemia (**g**) were recorded, CTRL, *n* = 12–40; OB, *n* = 11–72; and WL, *n* = 11–26, represented as Tukey boxplots (dots: outliers). Percentage of weaned pups per litter is indicated in (**h**), CTRL, *n* = 33; OB, *n* = 51; and WL, *n* = 21. A Principal Component Analysis on body weight and organ relative weights at sacrifice shows a separation of OB from CTRL and WL dams on dimension 1 (**i**), CTRL *n* = 16; OB, *n* = 8; and WL, *n* = 12.

As expected, dams under HFD had a greater caloric intake than dams under CD (Figure 2a). As a result, dams of the obese group (OB) were heavier than those of the control group (CTRL) at each time point from week 5.5 onwards, reaching +38% overweight before mating (all *p* < 0.05) (Figure 2b). Dams from the WL group were heavier than CTRL at each time point up to dietary intervention (all *p* < 0.05, Figure 2b). As soon as 3 days after switching HFD to CD and up to mating, WL females weighted less than OB (all *p* < 0.05). Nevertheless, WL dams remained +5% heavier than CTRL in the preconception period and at mating (all *p* < 0.05) (Figure 2b). Diet explained 25% of the variance in weight at week 5.5 (half a week after HFD exposure) and 61% of the variance at week 22 (4 months of diet). Interestingly, caloric intake relative to body weight was similar in the three groups at most time points in the preconception period (all *p* > 0.05), indicating a normal regulation of feeding behaviour of HFD-fed dams (Figure 2c). After 2 months under HFD, OB and WL dams were hypercholesterolemic, hyperleptinemic, hyperglycemic and glucose intolerant (all *p* < 0.05, see Figure 2d–g). At mating, OB dams conserved these disturbances (all *p* < 0.05), while all WL dams normalize these metabolic parameters except leptinemia (all *p* > 0.05 vs. CTRL, see Figure 2d–g). There was no extreme- or non-respondent WL individual.

### 3.2. Pup Survival Is Impaired by Maternal Obesity and Normalized by Maternal Weight Loss

The average crossing time (CTRL 3.3 ± 2.2 days; OB 2.8 ± 1.8 days; WL 2.9 ± 1.7 days, Kruskall–Wallis test *p =* 0.8) and the percentage of gestation loss (CTRL 6%; OB 4%; WL 9%, logistic regression *p =* 0.71) were similar for the three groups of dams. Gestation time was similar between groups (CTRL 19.5 ± 0.6 days; OB 19.8 ± 0.9 days; WL 19.4 ± 0.5 days; Kruskall–Wallis test, *p =* 0.09). We observed that the maternal group had a major effect on mortality of pups during the first postnatal week: CTRL and WL dams had more litters where at least one pup was weaned, compared with OB dams (48% for CTRL, 24% for OB and 67% for WL). Moreover, OB dams weaned fewer pups per delivered litter than CTRL or WL females (Figure 2h; *p =* 0.00017; post hoc test: OB vs. CTRL *p =* 0.017; WL vs. CTRL *p =* 0.39; WL vs. OB *p =* 0.0002). At sacrifice, one week after weaning, OB dams had an increased Sc, Pg, Pr and total WAT’s relative weight (*p* < 0.001), and a decreased kidney’s, heart’s and liver’s relative weight than CTRL dams (*p* < 0.001; Table S1). There was no difference between CTRL and WL. Principal Component Analysis was performed on all maternal parameters measured on dams after weaning at sacrifice (weight, WAT and BAT, kidneys’, heart’s and liver’s relative weights). CTRL and WL dams clustered together, away from OB dams on dimension 1 that explained 68.5% of the variance (Figure 2i).

Therefore, maternal obesity was associated with an increased pup mortality, while preconceptional WL normalized offspring survival.

### 3.3. Early Post-Natal Growth Curve Is Normalized by Maternal Preconceptional Weight Loss

**Figure 3:**
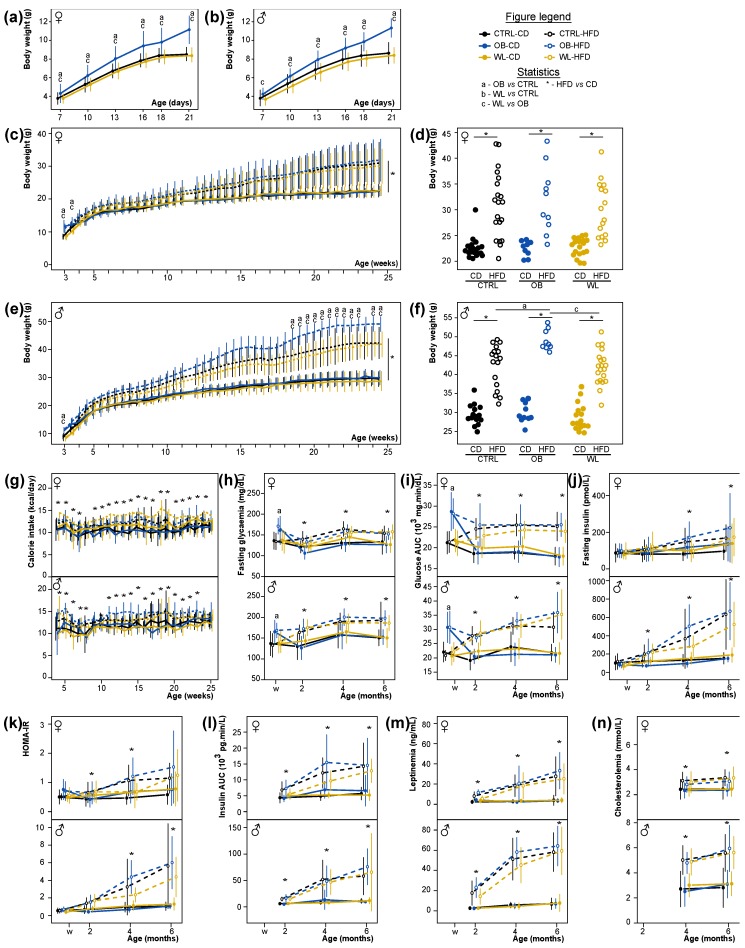
Male offspring born to OB mothers develop increased diet-induced obesity, WL offspring are not distinguishable from CTRL. F1 weight during the lactation period (**a**), female and (**b**), male, *n* = CTRL F:43, M:36, OB F:20, M:19, WL F:36, M:37) and evolution of body weight from weaning to 6 months of age ((**c**), female and (**e**), male, *n* = CTRL-CD F: 16–20, M: 12–15, CTRL-HFD F: 12–17, M: 13–17, OB-CD F: 7–10, M: 8–11, OB-HFD F: 7–11, M: 7–10, WL-CD F: 15–19, M: 11–17, WL-HFD F: 13–16, M: 14–20). Scatter plot of weight at week 24.5 to illustrate the distribution of the data ((**d**) females and (**f**) males). Evolution of food intake after weaning (**g**), fasting glycemia (**h**), OGTT glucose AUC (**i**), fasting insulinemia (**j**), HOMA-IR (**k**), OGTT insulin AUC (**l**), leptinemia (**m**), cholesterolemia (**n**) at weaning, 2, 4 and 6 months of age, represented as mean ± SD, *n* = CTRL-CD F: 13–20, M: 8–16, CTRL-HFD F: 13–21, M: 5–17, OB-CD F: 5–9, M: 8–10, OB-HFD F: 7–10, M: 7–9, WL-CD F: 7–19, M: 5–17, WL-HFD F: 6–17, M: 8–20 depending on age and investigated parameters.

We previously showed that maternal obesity induced foetal growth restriction (FGR: −13%) and increased the proportion of SGA foetuses (+28%) at term of gestation. This effect was corrected in WL dams [40]. As the pup’s survival was impaired in the early postnatal period, we only weighed the offspring starting from PND7 to avoid maternal abandonments. During the pre-weaning period, the offspring born to OB dams put on weight faster than CTRL (β = 0.13, *p* < 2.2 × 10^−16^). Maternal group influenced the male and female offspring weight, with OB differing from both CTRL and WL, except for PND7 males, where OB only differed from WL (Figure 3a,b).

### 3.4. Maternal Obesity Has Few Persistent Effects after Weaning and Preconceptional Weight-Loss Does Not Induce a Specific Metabolic Phenotype in the Offspring

After weaning, half of the offspring was fed a HFD and the other half a CD (Figure 1). The pre-weaning effects of the maternal group quickly disappeared and the F1 own post-weaning diet explained the majority of the metabolic phenotypes. For females, mice fed a HFD gained more weight than animals fed a CD as expected (β = 0.42, *p =* 2.9 × 10^−14^, Figure 3c). OB offspring were heavier than CTRL offspring at weeks 3 (*p =* 2.0 × 10^−12^) and 3.5 (*p =* 0.032, Figure 3c) and gained weight slower than CTRL from week 3 to 6 (β = −0.65, *p* < 0.001, Figure 3c). From week 6 to 25, there was no difference in growth curves and adult weight was not different according to maternal groups in F1 females (*p =* 0.75; Figure 3c,d). HFD-fed males also gained more weight than CD-fed as expected (β = 0.65, *p* < 2.2 × 10^−16^). OB males were heavier than CTRL males at week 3 (*p =* 0.000056, Figure 3e) and gained weight slower than CTRL until week 6 (β = −0.84, *p* < 0.001, Figure 3e). In F1 males only, there was an interaction between post-weaning diet, maternal group and time, with OB-HFD males gaining weight faster than CTRL-HFD males (β = 0.28, *p =* 0.01, Figure 3e). This interaction became significant after 18.5 weeks (all raw *p*-values < 0.05): OB-HFD males were 10.5% heavier than CTRL-HFD (Figure 3e,f). There was no difference in body weight between F1 born to WL and CTRL dams. The OB maternal effect was modest compared to the effect of the post-weaning diet, as maternal group explained only 2% of the variance at 6 months of age. However, among the HFD-fed male offspring, maternal group explained 26% of the variance in body weight.

HFD-fed female and male offspring ate more calories than CD-fed (*p* < 0.05 for most time points, Figure 3g), with no additional effect of maternal group. At weaning, male and female OB F1 had higher fasting glucose (*p =* 2.6 × 10^−11^, Figure 3h), and presented impaired glucose tolerance (higher AUC in OGTT, *p =* 3.48 × 10^−26^) compared with CTRL F1 (Figure 3i). For all parameters, WL offspring did not differ from CTRL animals. After weaning, the main effect is the own individual diet, with HFD-fed mice showing hyperglycemia, increased insulinemia and insulin resistance (HOMA-IR), decreased glucose tolerance (AUC), hypercholesterolemia and hyperleptinemia measured at 2, 4 and 6 months (Figure 3h–n), pointing at an impairment of glucose and lipid metabolism. The maternal group had no more effect on glucose and lipid metabolisms, nor on the evolution of the parameters between 2 and 6 months of age in both sexes (Figure 3h–n). A noticeable interaction between maternal group and post-weaning diet was visible at month 4 for fasting glycaemia in female, with WL-CD close to HFD-fed mice levels (*p =* 0.046, Figure 3h), for fasting insulin in males with WL-HFD close to CD-fed mice levels (*p =* 0.0028, Figure 3j) and for HOMA-IR, with WL-HFD close to CD-fed values (*p =* 0.043 in female and *p =* 0.004 in males, Figure 3k). However, this effect was not accompanied by glucose intolerance in month 4, as there was no statistical interaction in OGTT AUC measures, and was transitory, as it was no more observed at 6 months of age. Body composition at sacrifice was evaluated. Adiposity (Sc, Pg, Pr and total WAT) was increased under HFD in both sexes as expected (Table S1). Body composition was not affected by maternal group in F1 females (Table S1). In F1 males, there was an effect of maternal group on kidneys’ and heart’s weight: OB F1 had heavier kidneys and heart than WL (*p =* 0.001 and *p =* 0.0061 respectively; Table S1).

### 3.5. Post-Weaning HFD Offspring Display Poor Olfactory Performance

At 2 months of age, a hidden-cookie test was performed to measure the effect of maternal group and post-weaning diet on olfactory sensitivity. Maternal group, post-weaning diet and sex did not influence the offsprings’ digging activity towards a non-scented tile square, neither in fed state at the beginning nor in fasted state at the end of the experimental procedure.

**Figure 4:**
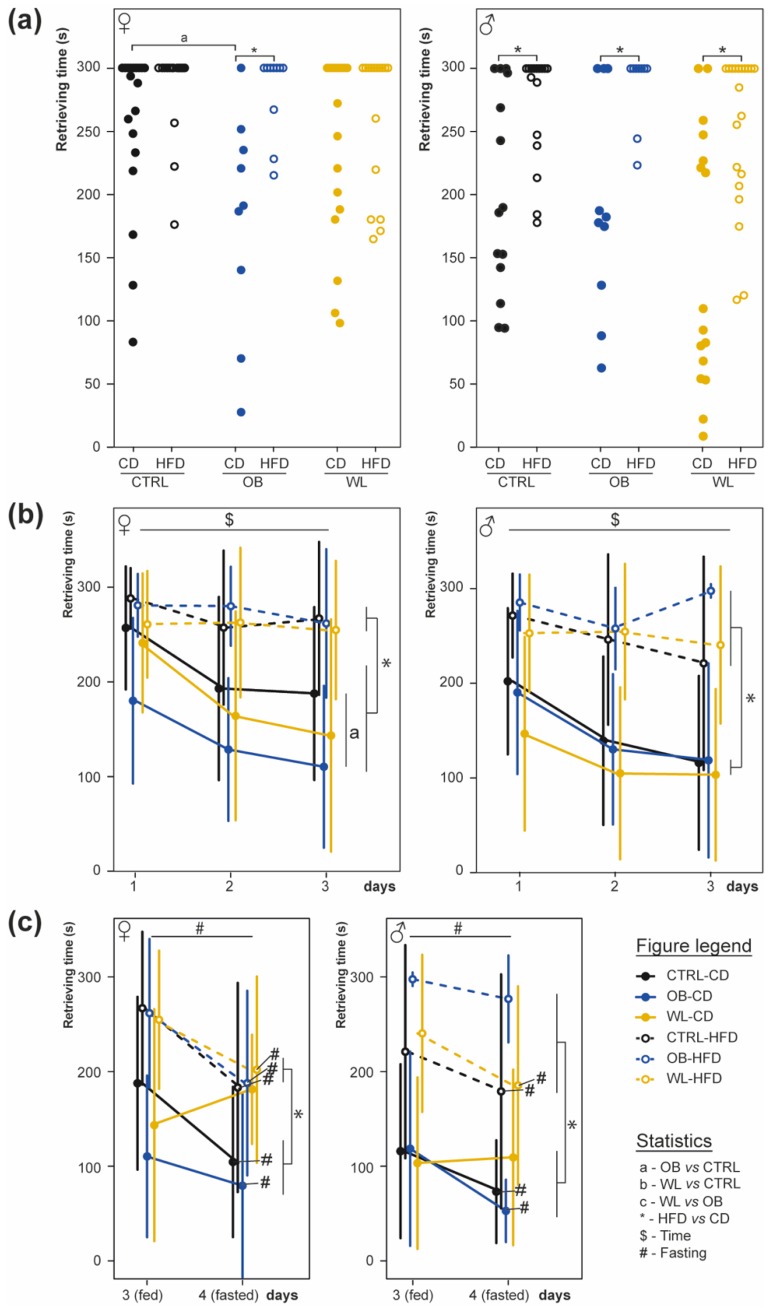
High fat post-weaning diet decreases mice performances in a general anosmia test. The hidden-cookie test measured the effect of maternal and post-weaning diet on general olfactory acuity at 2 months, after 4–5 weeks of post-weaning diet. The test was performed for 3 days (two averaged trials per day) in a fed state and on the 4th day after 8-h fasting. Latencies to find the buried cheese on the first day (**a**), their evolution between day 1 and 3 (**b**) and between day 3 (fed) and 4 (fasted) (**c**) are presented as scatterplots or mean ± SD for female (left; CTRL-CD *n* = 19, CTRL-HFD *n* = 20, OB-CD *n* = 9, OB-HFD *n* = 10, WL-CD *n* = 18, WL-HFD *n* = 16) and male (right; CTRL-CD *n* = 15, CTRL-HFD *n* = 16, OB-CD *n* = 10, OB HFD *n* = 9, WL-CD *n* = 16, WL-HFD *n* = 20) mice.

The time required to find the piece of cheese under the litter was analysed on the first experimental day (D1; Figure 4a). In female offspring, both maternal group (*p =* 0.03) and post-weaning diet (*p =* 0.0005) influenced retrieving time, with an interaction between factors (*p =* 0.04; Figure 4a left). HFD increased retrieving time in mice born to OB mothers (OB-CD vs. OB-HFD, *p =* 0.01; CTRL-CD vs. CTRL-HFD *p =* 0.12; WL-CD vs. WL-HFD, *p =* 0.4). Furthermore, CD mice born to OB dams displayed faster retrieving times (CTRL-CD vs. OB-CD *p =* 0.03). This effect was not observed in WL group (OB-CD vs. WL-CD, *p =* 0.5). In males, only post-weaning diet affected retrieving time (*p =* 2 × 10^−16^, Figure 4a right). Therefore, post-weaning diet highly influenced naive mice olfactory performance to localize the piece of cheese and this effect was modulated by maternal diet in female mice, the OB-CD females were indeed the best-performing group at this test.

The evolution of olfactory performance was evaluated by repeating the test three days in a row (Figure 4b). For both sexes, there was an effect of day (females, *p =* 0.004; males, *p =* 0.0003) and post-weaning diet (females, *p =* 5 × 10^−16^; males, *p =* 8 × 10^−24^), with an interaction between these factors (females *p =* 0.049; males *p =* 0.029). CD-fed mice displayed shorter retrieving time and improved their performance over time (*p =* 2.7 × 10^−4^), while HFD-fed mice did not (*p =* 0.47). In females, there was also an effect of maternal group (females, *p =* 0.042; males *p =* 0.17) and interaction between maternal group and post-weaning diet (females, *p =* 0.039; males *p =* 0.39). Concerning CD-fed mice, OB females were more performant than CTRL (*p =* 4 × 10^−4^), but there were no WL vs. OB and CTRL vs. OB differences (*p =* 0.1 and 0.06 respectively, Figure 4b). There were no between-group differences in HFD-fed females (*p =* 0.8, 0.4 and 0.33 respectively). Post-weaning HFD thus impaired olfactory learning/memory performance between D1 and D3 and, in females, OB mice fed a CD were the best-performing.

On the D4, we tested whether fasting could improve chemosensory performances depending on the group (Figure 4c). In females, there was no effect of maternal group, but an effect of both the post-weaning diet (*p =* 3.5 × 10^−8^), fasting (*p =* 3 × 10^−9^), as well as an interaction between maternal group and fasting (*p =* 4 × 10^−3^) and between post-weaning diet and fasting (*p =* 6 × 10^−3^, Figure 4c left). Fasting improved performance in CTRL-CD, CTRL-HFD, OB-CD, OB-HFD and WL-HFD (*p =* 2 × 10^−3^, *p =* 2 × 10^−4^, *p =* 0.05, *p =* 0.03, *p =* 0.006 respectively), but not WL-CD (*p =* 0.09). In males, a post-weaning diet and fasting effects were observed (*p =* 4 × 10^−16^ and *p =* 6 × 10^−7^), as well as an interaction between maternal group and post-weaning diet (*p =* 0.03, Figure 4c right). Fasting shortened retrieving time in CTRL-CD, CTRL-HFD, OB-CD and WL-HFD (*p =* 0.02, *p =* 0.06, *p =* 0.03, *p =* 0.02 respectively), but not in OB-HFD and WL-CD (*p =* 0.25 and *p =* 0.1). In both sexes, fasted mice on HFD still displayed the worse performance regardless of maternal group (Figure 4c) but fasting had no effect on WL-CD mice.

### 3.6. Post-Weaning Diet Influences Odour Habituation Capacities in Females

**Figure 5:**
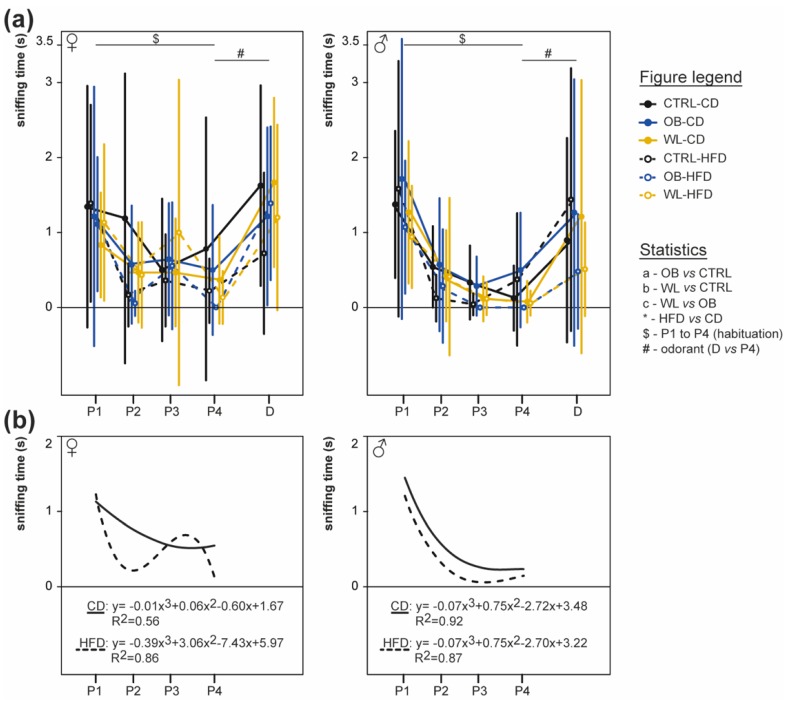
Olfactory discrimination is influenced by maternal and post-weaning diets. The habituation/discrimination test measured the ability to discriminate between a sequentially presented set of odours (four successive presentations of pentanol (P1–P4), followed by decanal (D)) at 5.5 months, after 18 weeks of CD or HFD diet. The exploration time for mice in response to the odorant presentation is given as mean ± SD (**a**) for female (left; CTRL-CD *n* = 17, CTRL-HFD *n* = 18, OB-CD *n* = 7, OB-HFD *n* = 9, WL-CD *n* = 15, WL-HFD *n* = 15) and male (right; CTRL-CD *n* = 11, CTRL-HFD *n* = 12, OB-CD *n* = 7, OB-HFD *n* = 7, WL-CD *n* = 12, WL-HFD *n* = 17). Regression curves for CD-fed and HFD-fed mice during habituation (P1–P4; (**b**)) for females (left) and male (right).

At 5.5 months of age, we tested whether maternal group or post-weaning HFD influences the habituation of mice to four repeated presentations of pentanol (Figure 5). In both sexes, there was no effect of the maternal group (female *p =* 0.96; male *p =* 0.66). In females, there was a global effect of odorant repetition (*p =* 3 × 10^−14^) and an interaction between odorant presentation and post-weaning diet (*p =* 0.03, Figure 5a left). Both CD- and HFD-fed mice habituate (CD, *p =* 0.004; HFD, *p =* 1 × 10^−5^), but CD-fed mice showed a continuous diminution of sniffing time between P1 and P4, whereas HFD-fed mice showed a slight increase at P3 (Figure 5b left). In males, there was an effect of odorant repetition (*p* = 2 × 10^−30^) and a post-weaning diet effect (*p* = 0.005, Figure 5a right): all males displayed odorant habituation and the HFD fed males sniffed less that CD (Figure 5b right).

As all animals displayed odorant habituation whatever their sex, maternal group or post-weaning diet, we tested the mice’s ability to discriminate two odorants by presenting a new odorant (decanal) as fifth presentation. Comparative analysis of sniffing time between the fourth pentanol and the decanal presentation showed that mice globally discriminated between these two odorants (females *p =* 2 × 10^−16^; males *p =* 2 × 10^−16^ Figure 5a), with neither effect of maternal group (females *p =* 0.94; males *p =* 0.64) nor post-weaning diet (females *p =* 0.08; males *p =* 0.19).

Therefore, post-weaning diet had sex-specific effects on odorant habituation.

### 3.7. Maternal Weight Loss Decreases the Amplitude of the Electrical Response of Olfactory Epithelium to a Non-Food Odorant

**Figure 6:**
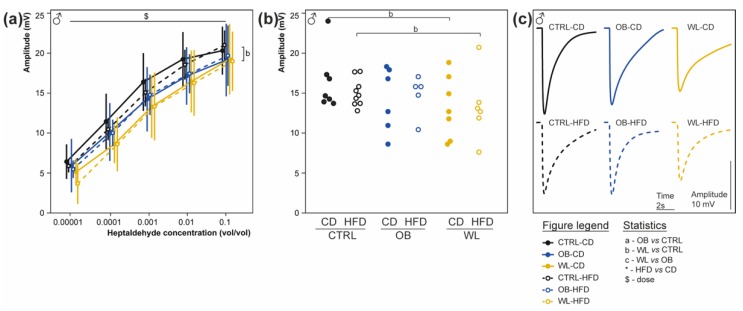
Weight loss decreases the electrical response of the olfactory epithelium to heptaldehyde in male. Electroolfactograms (EOG) recordings were obtained from olfactory epithelium (OE) from male mice at sacrifice at 6 months, after 23–25 weeks of post-weaning diet. The amplitudes of the EOG responses after successive application of increasing concentrations of HEP on both OE turbinates IIb and III are represented as mean ± SD for male mice ((**a**): CTRL-CD, *n* = 7; CTRL-HFD, *n* = 9; OB-CD, *n* = 6, OB-HFD, *n* = 5, WL-CD, *n* = 7 and WL-HFD, *n* = 6). Scatterplot representation (**b**) and typical trace (**c**) of EOG responses to heptaldehyde at 1:1000 in mineral oil are given for the 6 groups.

At 6 months of age, we performed EOG recordings on male’s olfactory epithelium (OE) to analyse the amplitude of electrophysiological responses to the application of increasing heptaldehyde (HEP) concentrations from the 6 groups (Figure 6). Amplitude of the OE electrical response increased with the odorant concentration for all groups (*p =* 2 × 10^−16^). Not the post-weaning diet (*p =* 0.74), but the maternal group had an impact on the responses to odorants (*p =* 0.012), with no interaction (Figure 6). Offspring born to WL mothers displayed decreased response amplitudes to HEP compared with offspring born to CTRL (WL CD vs. CTRL-CD, *p =* 0.014; WL-HFD vs. CTRL-HFD, *p =* 0.005), but not compared with offspring born to OB (WL-CD vs. OB-CD *p =* 0.38; WL-HFD vs. OB-HFD *p =* 0.16). There was no difference between OB and CTRL. Therefore, there was a decrease in the olfactory sensitivity for a non-food odorant of offspring born to WL dams, whatever the post-weaning diet.

### 3.8. Post-Weaning Diet Has a Major Effect on F1 Phenotype and HFD-Fed Males Are Additionally Influenced by Maternal Group

**Figure 7:**
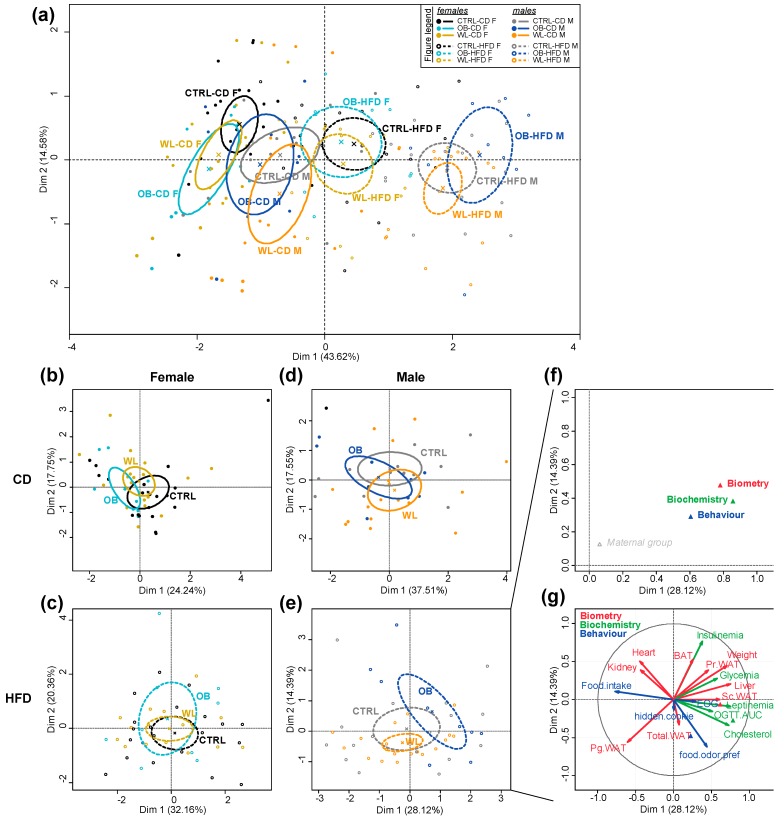
Multiple factor analysis (MFA) reveals an influence of maternal group on HFD-fed males. “Biometry”, ”Biochemistry” and “Behaviour” data from females and males were analysed by MFA. Plot ellipses with their barycentre for maternal group are represented on the two first dimensions of the MFA for all the offspring (**a**), females (**b**,**c**) and males (**d**,**e**) and according to post-weaning diet CD (**b**,**d**) or HFD (**c**,**e**). Correlation (*r^2^*) of the variables groups to the two dimensions of the MFA is represented on the categories correlation graph (**f**) and correlation (*r*) of the variables to the two dimensions of the MFA on the variables factors map (**g**) for HFD-fed males.

To get a global overview of the effect of maternal group on offspring health, we performed a multiple factor analysis (MFA) on all the data previously described. Variables were separated into three classes: “Biochemistry”, “Biometry” and “Behaviour”. In line with the previous results, there was a major effect of sex and post-weaning diet (Figure 7a). We therefore performed the MFA on mice split by sex and post-weaning diet to better reveal the effect of maternal group. While there was no effect of the maternal group neither in females (Figure 7b,c) nor in CD-fed males (Figure 7d), maternal groups separated on the HFD-fed male chart (Figure 7e). A hierarchical clustering of the individuals was performed and showed a difference between maternal groups (χ^2^ test, *p =* 0.0234), with an anti-correlation of the OB group (v.test = −2.533; *p =* 0.011). Separation between OB and WL offspring occurred on the two first dimensions (42.5% of the variability) with an association (OB *p =* 0.044; WL *p =* 0.047) of these maternal groups to the second dimension (14.4% of the variability; Figure 7e). The most representative variable group to the first dimension was “Biochemistry” (*r²* > 0.8) and the second dimension was represented by “Biometry” (*r²* > 0.45; Figure 7f). “Behaviour” was mostly represented in the third dimension (*r²* > 0.6) and contributed to the differences in the first dimension (*r²* > 0.6). On the first dimension of the graph of variables (28.1% of the variability), OB offspring were associated to variables leptinemia, liver relative weight, cholesterolemia, body weight and Sc WAT (*p* < 1 × 10^−5^), while WL offspring were associated with variables food intake and Pg WAT (*p* < 1 × 10^−5^) (Figure 7g). On the second dimension, OB offspring were associated with insulinemia (*p* < 7 × 10^−10^) and WL offspring with food odour preference (*p* < 6 × 10^−6^) (Figure 7g).

Therefore, in both sexes, post-weaning diet had a major effect. More interestingly, the maternal weight trajectories influenced the offspring’s phenotype in HFD-fed males.

## 4. Discussion

We investigated the effects of a maternal obese environment and preconceptional WL on the evolution of selected metabolic measures over time, along with obesity-induced olfactory phenotypes in offspring. We found that:

1. The main effects on metabolic and olfactory phenotypes are linked to the post-weaning diet.
2. There is a noticeable influence of the maternal group: OB-HFD are more susceptible to develop obesity than CTRL-HFD males, OB-CD females are the best performing in the cookie test, fasting does not improve the performance of WL-CD. In HFD-fed offspring, MFA reveals differences between OB and WL mice.
3. While maternal WL has positive effects on offspring metabolism, there is a programming effect of the preconceptional intervention on olfactory sensitivity.
4. Sex differences are observed: maternal obesity impairs male metabolism, and improves olfactory performances in female fed a CD. Odorant habituation is sensitive to the postweaning diet in female only.

To our knowledge, this is the first study addressing the respective role of preconceptional/perinatal and postweaning nutrients modulation on combined metabolic and olfactory outcomes, both known to contribute to food intake regulation, with a special focus on sex differences.

### 4.1. Post-Weaning Diet Determined Metabolic and Olfactory Behavioural Risk in Mice

As expected, HFD post weaning was a major contributor of both metabolic and olfactory disturbances. It indeed explains more than 45% of the variance in MFA, and was in line with other studies on metabolic outcomes of HFD [46]. Interestingly, HFD was associated with a low motivation and learning capacities to find a food reward after 5 weeks of diet only, yet sensitive to fasting in both sexes. However, later in adulthood, HFD-fed female mice even better performed in the habituation task, and no significant change in EOG response was noticed in males under HFD at sacrifice. The absence of long-term effect is quite unexpected regarding the effects in juvenile mice, but experiments were performed several months apart, based on different stimuli, sensitivities and neural pathways. Accordingly, a short-term effect of maternal obesity on juvenile male olfactory performances has been recently described, but was not associated to a modification of EOG response [18]. Other behavioural studies have reported variable long-term effects of genetic obesity [4,47,48] or diet [5,6,49] on olfactory performances in animals and humans [38,50,51]. Therefore, the deleterious impact of obesogenic context, either in the perinatal or post-weaning period, more likely derives from a modification in the central olfactory processing. We suggest that, rather than a long-term modification in the olfactory sensitivity, HFD might have influenced animal motivation to retrieve and consume the appetitive reward after few weeks of HFD. Indeed, diet-induced obesity induces a substantial decline in motivated behaviour, as for appetitive learning tasks [52,53] or responses to palatable rewards [54,55] as well as impairments in memory, attention or reward (for reviews, see [56,57]), some of these cognitive disorders being observed rapidly after diet change [18,58,59]. Finally, contrary to our hypothesis, we did not observe any long-term effect of an early HFD context on olfactory preference for familiar energy-rich pellets later, as published for food preferences [20,60]. Moreover, this was also observed recently for maternal high-protein diet, where only the F1 own diet influenced macronutrient self-selection [19].

This suggests that disturbance of olfactory sensitivity or cognitive function, or both, could develop along with metabolic imbalance in juvenile age in a maternal lipid-rich context, thus possibly fueling the vicious circle of appetite dysregulation and unhealthy food or nutrients choices [57]. Our findings thus favour a susceptibility to HFD exposure regarding offspring behavioural outcomes at adolescence, a period of neurobehavioural shaping preparing lifelong cognitive processing from the olfactory tract to the more central regions [61,62,63].

### 4.2. Maternal Obesity Affects Male Offspring Weight and Male and Female Olfactory Behaviour

We observed only a weak effect of maternal obesity on both metabolic and behavioural phenotypes. We previously reported that maternal obesity leads to an increased SGA phenotype at term of gestation [40]. Here, we report an increased risk for obesity in adult HFD-fed males born to OB mother. We also noticed that CD-fed females born to OB mothers better performed compared to those born to CTRL mothers in the hidden cookie test, suggesting that maternal obesity could improve olfactory and/or cognitive functions, providing that animals are on a healthy diet. As already discussed for HFD, the maternal OB context might have influenced offspring neurocognitive performances by enhancing motivation for fat-rich reward [20,60,64,65].

From our previous results of increased SGA phenotype in this model [40] and from the literature [66,67,68], we expected stronger effects of maternal obesity. Several publications have recently pointed a small or inexistent impact of maternal obesity on offspring metabolic profile in mice [46,69,70]. There could rather be associations to some neurocognitive effects such as enhanced hedonic-like behaviours [64], not tested in our behavioural analysis. Other studies suggest the need for a mismatch of pre-pregnancy CD and gestational HFD to program glucose intolerance in offspring [71]. A recent meta-regression analysis concluded that maternal HFD exposure affects metabolic parameters in the offspring, but also underlined significant bias toward the non-publication of ‘negative’ results [72]. Finally, in our model, the modest survival of pups from OB mothers during the first week of life may have limited the expression of a maternally induced phenotype. The selected F1 survivors, less affected by maternal environment, may be less prone to develop metabolic or behavioural disturbances.

### 4.3. Maternal Weight Loss Normalized Metabolic Parameters But Led to Impaired Olfactory Sensitivity

We previously showed that preconceptional WL was beneficial to foetal growth, with a partial normalization in placental and hepatic gene expression patterns [40]. This suggests that maternal obesity, even when corrected before conception, leaves a “metabolic footprint”. In the present long-term study, maternal preconceptional WL normalized adult offspring weight and was not associated with any deleterious metabolic profile, but the MFA performed on all combined phenotypes showed a separation between WL and OB male offspring. Particularly, WL male offspring exhibited a lower peripheral olfactory sensitivity, and fasting did not improve motivation to retrieve cheese in WL mice under CD. As EOG were performed in males only, we cannot exclude possible altered mucosal sensitivity in females as well. As already reported in various rat and sheep models [33,34,35,36], the nutritional intervention did not entirely restore maternal preconceptional weight and female remained slightly heavier than controls. In the offspring, some phenotypes were not normalized at all, or were at an intermediate level between obese and control mothers.

The decrease in olfactory sensitivity in WL males and the resistance of WL-CD to fasting could stem from the preconceptional caloric depletion associated with the transition to CD in mothers or to a maternal stress linked to the procedure of food transition per se, through the rapid lost weight. Indeed growing evidence suggests that preconceptional adversity may affect the progeny even in adulthood [73]. Interestingly, a nutritional intervention could increase the offspring hypothalamo-pituitary-adrenal axis response, independently from the mother’s metabolic status [35,74]. Female offspring born to moderate calorie-restricted mothers displayed increased vulnerability in the olfactory bulb and in regions involved in feeding control [17]. Therefore, maternal nutritional stress might have altered the neurocognitive development of the offspring. Besides, the low motivation of fasted WL-CD mice to find hidden cheese could illustrate the expression of an olfactory-guided behaviour in a non-normalized metabolic context. Indeed, while fasting improved smelling capacity in lean animals, obese animals usually remain unsensitive to fasting [47,49,75]. Further studies are needed to discriminate the effect of energy depletion from the effect of stress on the maternal effects reported herein.

### 4.4. Sex Differences in the Offspring Phenotype

Our data pointed out a noticeable male-preferential impact in the body weight conditioning by maternal obesity, as it was previously reported [76]. Accordingly, the MFA analysis summarized this sex-specific outcome. Maternal effect on olfactory phenotypes was instead stronger in female: OB-CD were the best performing in the hidden cookie test and differed in habituation profile to pentanol according to post-weaning diet. Sex specificities occur in most NCDs, but it is recent that animal models in the DOHaD context do not only focus on male offspring. Disparity between males and females includes the timing of onset and severity of metabolic disease outcomes and neurobehavioural changes in response to maternal metabolism [17,77]. In the meta-analysis cited above, maternal HFD was associated with increased body weight, adiposity, dyslipidaemia and insulinaemia in all mice but hyperglycaemia was restricted to females [72]. Moreover, some sex-divergent behavioural consequences of HFD have been attributed to a differential activation of the neuroendocrine system [78]. Female olfactory performances have not however been studied widely. To our knowledge, this is the first study that includes males and females in the same cohort.

## 5. Conclusions

In conclusion, we demonstrated that maternal preconceptional weight loss roughly normalized the survival and metabolic profile of the offspring, but induced a reduction in the sensitivity of the olfactory mucosa. Maternal weight loss was not associated to any other deleterious phenotype. It remains unclear whether the programming effect of maternal weight loss is due to maternal stress or nutrition per se.

In humans, preconceptional weight loss is obviously recommended to obese women, yet few studies have been conducted on the consequences for the offspring. We believe that more animal experiments and human cohorts are needed to clarify the beneficial effect of maternal preconceptional weight loss on the offspring’s health.

## Figures and Tables

**Table 1:** Diet composition of the CD and HFD formulas (Research Diets, New Brunswick, NJ, USA).

Formula	CD		HFD	
	#D12450K		#D12492	
Composition	g	kcal	g	kcal
Protein	19.2	20%	26.2	20%
Carbohydrate	67.3	70%	26.3	20.1%
Fat	4.3	10%	34.9	59.9%
Total		100%		100%
Energy (kcal/g)	3.85		5.24	
Ingredient	g	kcal	g	kcal
Casein, 80 Mesh	200	800	200	800
L-Cystine	3	12	3	12
Corn starch	550	2200	0	0
Maltodextrin 10	150	600	125	500
Sucrose	0	0	68.8	275.2
Cellulose, BW200	50	0	50	0
Soybean oil	25	225	25	225
Lard	20	180	245	2205

CD, control diet; HFD, high-fat diet.

## Supplementary Materials

The following are available online at https://www.mdpi.com/2072-6643/11/5/948/s1, Methods S1: Food odour preference test. Figure S1: Food odour preference is not influenced by post-weaning diet but may be influenced by maternal group. Table S1: Body composition of mothers at sacrifice (1 weak post-weaning) and offspring at sacrifice (6 months of age) after 6-h fasting.
