# Supplementary material for: Effect of Maternal Obesity and Preconceptional Weight Loss on Male and Female Offspring Metabolism and Olfactory Performance in Mice
Source: Nutrients. 2019 Apr 26;11(5):948. doi: 10.3390/nu11050948 (PMC6566604; doi:10.3390/nu11050948)
Supplement: Supplementary file 1 [file nutrients-11-00948-s001.pdf]

Effect of maternal obesity and preconceptional weight loss on male and female offspring metabolism and olfactory performance in mice

Supplementary Materials

Polina E. Panchenko[1]*, Marie-Christine Lacroix[2]*, Mélanie Jouin[1], Sarah Voisin[1], Karine Badonnel[2], Marion Lemaire[1], Nicolas Meunier[2], Sofiane Safi-Stibler[1], Marie-Annick Persuy[2], Luc Jouneau[1], Didier Durieux[2], Simon Lecoutre[3], Hélène Jammes[1], Delphine Rousseau-Ralliard[1], Christophe Breton[3], Claudine Junien[1], Christine Baly[2] and Anne Gabory[1#].

* P.E. Panchenko and M-C. Lacroix have contributed equally to this work

Affiliations

[1] UMR BDR, INRA, ENVA, Université Paris Saclay, 78350, Jouy-en-Josas, France

[2] NBO, INRA, Université Paris-Saclay, 78350 Jouy-en-Josas, France

[3] Univ. Lille, EA4489, Équipe Malnutrition Maternelle et Programmation des Maladies Métaboliques, F-59000 Lille, France

[#] **corresponding author:** anne.gabory@inra.fr, tel: 00-33-1-34-65-23-48

Methods S1

Food odour preference test. A four hole-board was used, in a two opposite holes configuration. Animals were familiarized with the experimental setup by a 2 x 3 min trial with the glass container filled with the half tea-ball cover. Then two glasses containers in opposite position, one containing a pellet of CD and the second a pellet of HFD, each covered with a half tea-ball cover were introduced. Mice were placed on the middle of the board and the time spent sniffing CD- or HFD-filled containers was manually recorded for 3 min. The container positions were changed at each trial, but always in opposite position to optimize the spatial separation of both odours. Between each trial, animals returned in their cage for 5 min. Animals that did not display any sniffing during the test were removed from the statistical analysis (2 CTRL-CD and 2 CTRL-HFD males, 2 WL-CD and 2 WL-HFD females). The preference for HFD-filled hole was calculated as the ratio between the time spent sniffing the HFD and the total time sniffing. The preference for HFD-filled hole was tested with one sample t-test compared to the theoretical value of no choice at 50%. N-ways ANOVAs with 2 factors (*Groups*Diet*) followed by two-sample Fisher-Pitman permutation test, stratified by *cohort* and corrected by Benjamini and Hochberg, were performed to compare the preference ratio between group.

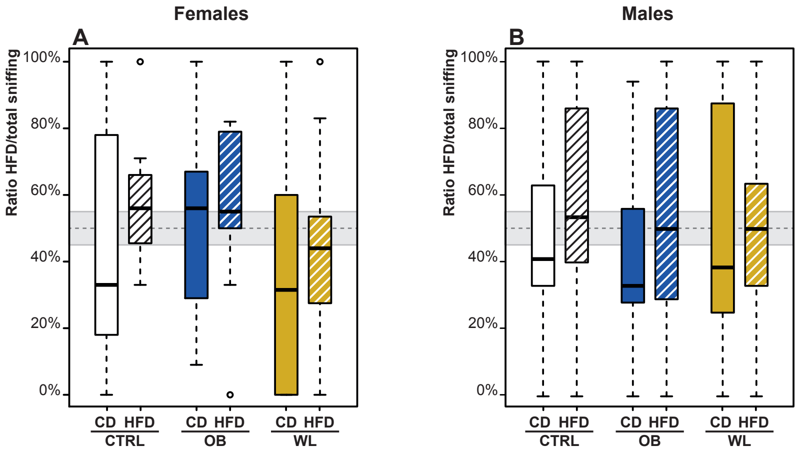

Figure S1: Food odour preference is not influenced by post-weaning diet but may be influenced by maternal group The time spent sniffing CD- or HFD-filled holes was recorded for 3 min in a 2 hole-board test on 6 hr-fasted F1 mice after 22 weeks of post-weaning diet. The preference for HFD-filled hole was calculated as the ratio between the time spent sniffing the HDF and the total time sniffing, presented as Tukey box plot for the 6 groups of female **(a;** CTRL-CD n=19, CTRL-HFD n=20, OB-CD n=9, OB-HFD n=10, WL-CD n=16, WL-HFD n=14) and male (**b**; CTRL-CD n=13, CTRL-HFD n=14, OB-CD n=10, OB-HFD n=9, WL-CD n=16, WL-HFD n=20) mice.

At 6.5 months, we tested whether maternal group or post-weaning diet influences the mice's level of interest for food odours, we analysed the sniffing time of CD and HFD pellets simultaneously presented to the animal in two separated holes. During the habituation phase, in absence of food odour, we observed no influence of maternal group, diet or sex on sniffing behaviour and no preference for a specific hole, with very low mean sniffing time (data not shown). After food odour introduction, there was no effect of maternal group (p=0.68 and p=0.71 for females and males, respectively) or diet (p=1 and p=0.2 for females and males, respectively) on the total time spent sniffing CD or HFD holes. There was no preference for the HFD-filled hole, regardless of maternal or post-weaning diet. We then tested whether there was a between-group difference in the ratio between the time spent sniffing HFD and total time. There was no effect of maternal group (females, p=0.73; males, p=0.09) or post-weaning diet and no interaction. Therefore, HFD-fed mice did not show any olfactory preference to either CD or HFD, and maternal group didn't influence the preference ratio.

	Weight (g)	Sc-WAT	Pg-WAT	Pr-WAT	Total-WAT	BAT	Kidneys	Heart	Liver
**Mother**									
CTRL, n=17	24.9(1.3)	2.16(6.44)	2.23(0.47)	0.71(0.23)	5.11(0.91)	0.37(0.10)	1.14(0.04)	0.58(0.04)	4.80(0.72)
OB, n=8	33.2(3.5)[a]	6.44(1.75)[a]	6.90(1.91)[a]	2.13(0.66)[a]	15.47(4.19)[a]	0.31(0.07)	0.97(0.15)[a]	0.43(0.03)[a]	3.50(0.57)[a]
WL, n=12	25.1(1.2)[b]	2.17(0.34)[b]	2.40(0.58)[b]	0.65(0.13)[b]	5.23(0.98)[b]	0.41(0.13)	1.18(0.07)[b]	0.59(0.04)[b]	4.45(0.63)[b]
ANOVA	$p<0.01$	$p<0.001$	$p<0.001$	$p<0.001$	$p<0.001$	NS	$p<0.001$	$p<0.001$	$p<0.001$
**Female offspring**									
CTRL-CD, n=20	22.3(2.1)	2.66(1.01)	2.61(0.79)	0.82(0.28)	6.09(1.96)	0.35(0.08)	1.13(0.16)	0.54(0.07)	3.65(0.48)
OB-CD, n=9	21.8(1.0)	2.03(0.51)	2.25(0.35)	0.75(0.17)	4.64(1.08)	0.33(0.11)	1.23(0.11)	0.58(0.05)	3.76(0.46)
WL-CD, n=19	21.7(1.8)	2.31(0.57)	2.49(0.63)	0.82(0.24)	5.62(1.31)	0.40(0.13)	1.10(0.14)	0.55(0.06)	3.62(0.52)
CTRL-HFD, n=20	30.4(6.4)	6.25(1.82)	7.42(2.57)	2.23(0.66)	15.90(4.71)	0.39(0.16)	0.91(0.16)	0.45(0.08)	2.83(0.32)
OB-HFD, n=10	31.6(6.1)	5.93(1.42)	7.49(1.82)	2.32(0.80)	15.15(3.76)	0.42(0.22)	0.97(0.06)	0.46(0.04)	2.73(0.35)
WL-HFD, n=15	29.0(5.1)	5.96(1.73)	6.93(1.83)	2.27(0.74)	15.17(3.97)	0.38(0.12)	0.91(0.12)	0.44(0.06)	2.73(0.40)
post-weaning diet	$p<0.01$	$p<0.01$	$p<0.01$	$p<0.01$	$p<0.01$	NS	$p<0.01$	$p<0.01$	$p<0.01$
maternal group	NS	NS	NS	NS	NS	NS	NS	NS	NS
**Male offspring**									
CTRL-CD, n=15	28.4(2.7)	2.51(0.80)	3.16(1.31)	1.32(0.56)	6.98(2.57)	0.51(0.15)	1.17(0.19)	0.53(0.08)	3.42(1.31)
OB-CD, n=9	29.0(2.8)	2.33(0.46)	3.10(1.09)	1.15(0.31)	6.58(1.72)	0.47(0.09)	1.26(0.17)	0.59(0.10)	3.47(0.69)
WL-CD, n=17	27.4(3.4)	2.49(0.76)	2.94(0.97)	1.27(0.76)	6.80(2.37)	0.49(0.16)	1.07(0.11)	0.51(0.05)	3.50(0.43)
CTRL-HFD, n=17	41.7(5.5)	6.75(0.94)	5.75(1.62)	3.34(0.62)	15.84(1.57)	0.70(0.27)	0.86(0.11)	0.41(0.07)	3.24(0.88)
OB-HFD, n=9	46.3(3.4)	7.51(1.41)	4.87(0.74)	4.02(0.69)	16.40(1.97)	0.87(0.47)	0.91(0.11)	0.42(0.08)	3.42(0.59)
WL-HFD, n=19	41.3(3.9)	6.58(0.99)	6.32(1.35)	3.08(0.62)	15.98(1.50)	0.57(0.24)	0.83(0.06)	0.37(0.03)	3.06(0.47)
post-weaning diet	$p<0.01$	$p<0.01$	$p<0.01$	$p<0.01$	$p<0.01$	$p<0.01$	$p<0.01$	$p<0.01$	NS
maternal group	NS	NS	NS	NS	NS	NS	$p=0.028$	$p=0.041$ ([c], $p=0.001$)	NS ([c], $p=0.006$)

<u>Table S1:</u> Body composition of mothers at sacrifice (1 weak post-weaning) and offspring at sacrifice (6 months of age) after 6-hour fasting. Weights of organs are presented as percentage of body weight, indicated as mean%(SD). ANOVA *p*-values are indicated or NS (non-significant), followed by Tukey's post-hoc tests for maternal group, indicated as [a]OB vs CTRL, [b]WL vs CTRL, [c]WL vs OB.
